# Underweight Patients with Low Bone Density are at Greater Risk of Intraoperative Fracture during Hemiarthroplasty

**DOI:** 10.1055/s-0045-1814124

**Published:** 2025-12-22

**Authors:** Jun Takeuchi

**Affiliations:** 1 Department of Orthopedic Surgery, Yonemori Hospital, Yojirou, Kagoshima City, Kagoshima, Japan

**Keywords:** body mass index, bone mineral density, femoral fractures, femoral neck fractures, risk factors, densidade mineral óssea, fatores de risco, fraturas do colo femoral, fraturas do fêmur, índice de massa corporal

## Abstract

**Objective:**

To investigate whether body mass index (BMI) modifies the association between bone mineral density (BMD) and intraoperative fracture risk in cementless hemiarthroplasty.

**Methods:**

Between April 2020 and March 2025, 588 patients who received cementless stems and underwent dual-energy X-ray absorptiometry scans within one week postoperatively were included. Patients were stratified by BMI (≥ 18.5 kg/m ^2^ versus < 18.5 kg/m ^2^ ) and young adult mean (YAM) (cutoff: 70%). Fracture risk was compared across groups. Subgroup analyses based on YAM levels and operative parameters were performed in 577 patients with complete data.

**Results:**

In underweight patients (BMI < 18.5 kg/m ^2^ ), lower YAM was associated with higher intraoperative fracture risk ( *p* = 0.128), whereas no significant association was observed in patients with BMI ≥ 18.5 kg/m ^2^ ( *p* = 0.80). Fracture rates increased with lower YAM: 3.97% (≥ 70%), 5.24% (60–69%), 5.88% (50–59%), 6.58% (< 50%), though not statistically significant. Higher BMI was associated with longer operative time and greater blood loss.

**Conclusion:**

Body mass index modifies the association between BMD and intraoperative fracture risk. Preoperative YAM assessment may help identify underweight patients at higher risk during cementless hemiarthroplasty. Level of Evidence III, therapeutic study

## Resumo

**Objetivo:**

Investigar se o índice de massa corporal (IMC) modifica a associação entre densidade mineral óssea (DMO) e risco de fratura intraoperatória em hemiartroplastia não cimentada.

**Métodos:**

Entre abril de 2020 e março de 2025, foram incluídos 588 pacientes que receberam hastes não cimentadas e foram submetidos a exames de raios X de dupla energia em até uma semana de pós-operatório. Os pacientes foram estratificados por IMC (≥ 18,5 kg/m ^2^ versus < 18,5 kg/m ^2^ ) e porcentagem média de adultos jovens (YAM, do inglês *young adult mean* ) (ponto de corte: 70%). O risco de fratura foi comparado entre os grupos. Análises de subgrupos, com base nos níveis de YAM e nos parâmetros operatórios, foram realizadas em 577 pacientes com dados completos.

**Resultados:**

Em pacientes com baixo peso (IMC < 18,5 kg/m ^2^ ), menor YAM associou-se a maior risco de fratura intraoperatória ( *p* = 0,128), enquanto nenhuma associação significativa foi observada em pacientes com IMC ≥ 18,5 kg/m ^2^ ( *p* = 0,80). As taxas de fratura aumentaram com menor YAM: 3,97% (≥ 70%), 5,24% (60–69%), 5,88% (50–59%), 6,58% (< 50%), embora esse aumento não tenha sido estatisticamente significativo. O IMC mais alto foi associado a maior tempo operatório e maior perda de sangue.

**Conclusão:**

O IMC modifica a associação entre DMO e risco de fratura intraoperatória. A avaliação pré-operatória do YAM pode ajudar a identificar pacientes com baixo peso e com maior risco durante a hemiartroplastia não cimentada. Nível de evidência III, estudo terapêutico.

## Introduction

Proximal femoral fractures are common injuries in elderly populations, representing a major public health burden worldwide due to the aging society. Hip arthroplasty is frequently performed for displaced femoral neck fractures, providing favorable functional outcomes and pain relief. However, intraoperative periprosthetic fractures remain one of the major complications during arthroplasty procedures, particularly in elderly and osteoporotic patients.

Bone mineral density (BMD) is considered a key factor influencing the risk of intraoperative fracture. Preoperative assessment of BMD, often expressed as a young adult mean (YAM) percentage, has been proposed as a useful parameter for evaluating bone quality in surgical candidates. In addition, body mass index (BMI), reflecting nutritional status and sarcopenic tendencies, may also play an important role in bone fragility and fracture susceptibility.

Although both BMD and BMI are recognized as important contributors to bone strength, limited studies have explored the potential interaction between these factors in the context of intraoperative periprosthetic fractures. Understanding how BMI modifies the association between BMD and intraoperative fracture risk may offer useful insights for surgical risk stratification and preoperative optimization.

The purpose of the present study was to investigate whether BMI modifies the predictive value of BMD for intraoperative periprosthetic fractures in patients undergoing hip arthroplasty for femoral neck fractures.

## Materials and Methods

### Study Design and Patient Selection

The present retrospective study was conducted at our institution between April 1, 2020, and March 31, 2025. During this period, 1,119 patients underwent hip arthroplasty for femoral neck fractures. Among them, 588 patients who received cementless femoral stems and underwent bone mineral density (BMD) assessment using dual-energy X-ray absorptiometry (DEXA) within 1 week postoperatively on the contralateral hip were included in the analysis. Patients with missing BMD data or incomplete clinical information were excluded.

### Data Collection

Bone mineral density was measured using DEXA with a Lunar Prodigy scanner (GE Healthcare). The regions of interest included the femoral neck, greater trochanter, and proximal shaft. Values were expressed as a percentage of the young adult mean (YAM), based on Japanese reference data for individuals aged 20 to 39 years.

The following demographic and clinical variables were collected from medical records: sex, age, laterality (right or left), body mass index (BMI), and BMD. Bone mineral density was expressed as a percentage of the young adult mean (YAM), which is commonly used in Japan as an indicator of bone quality.

### Group Classification

Patients were stratified into 4 groups according to international BMI classification (cutoff at 18.5 kg/m ^2^ ) and YAM threshold (cutoff at 70%). This created the following categories:

YAM ≥ 70% and BMI ≥ 18.5 kg/m ^2^ ;
YAM ≥ 70% and BMI < 18.5 kg/m ^2^ ;
YAM < 70% and BMI ≥ 18.5 kg/m ^2^ ; and
YAM < 70% and BMI < 18.5 kg/m ^2^ .

Additionally, patients were further categorized into 4 groups based on YAM levels alone:

YAM ≥ 70%;
YAM 60–69%;
YAM 50–59%; and
YAM < 50%.

### Outcome Assessment

The primary outcome was the occurrence of intraoperative periprosthetic fracture, which was assessed intraoperatively and confirmed by intraoperative findings and postoperative imaging.

### Statistical Analysis

In addition, subgroup analyses were performed in 577 patients with complete data for operative time and blood loss, to compare these operative parameters between patients with BMI ≥ 18.5 kg/m ^2^ and those with BMI < 18.5 kg/m ^2^ . Mann-Whitney U tests were used to assess statistical significance.

Descriptive statistics were used to summarize the patients' characteristics. Intraoperative fracture rates were compared among groups. Stratified analysis was performed to assess whether BMI modified the association between YAM and fracture risk. Fisher's exact test was used to calculate odds ratios (ORs) and *p* -values. A *p* -value of < 0.05 was considered statistically significant. Statistical analyses were conducted using standard software.

## Results

A total of 588 patients were included in the present study ( Table 1 ). The mean age was 83.8 ± 8.1 years, and there were 160 males and 428 females. The mean BMI was 20.19 ± 4.20 kg/m ^2^ , and the mean YAM was 62.4 ± 11.8%.

**Table 1:** Baseline characteristics stratified by YAM

Variable	YAM < 70%	YAM ≥ 70%
n	264	83
Mean age (years)	84.6 ± 7.7	81.7 ± 8.0
Female sex: n (%)	204 (77.3%)	54 (65.1%)
Mean BMI (kg/m ^2^ )	19.50 ± 3.36	21.55 ± 3.57
Mean YAM (%)	57.4 ± 8.0	76.0 ± 7.4
Mean operative time (minutes)	55.5 ± 18.1	58.7 ± 19.8
Mean blood loss (mL)	134.7 ± 110.2	124.1 ± 72.5
Fracture: n (%)	5 (1.9%)	3 (3.6%)

Abbreviation: YAM, young adult mean.

Baseline characteristics stratified by YAM are shown in Table 1 , and those stratified by combined YAM × BMI categories are presented in Table 2 . These tables provide the detailed distribution of demographic and operative variables across the analytical groups.

**Table 2:** Baseline characteristics stratified by YAM and BMI

Variable	YAM < 70% and BMI < 18.5 kg/m ^2^	YAM < 70% and BMI ≥ 18.5 kg/m ^2^	YAM ≥ 70% and BMI < 18.5 kg/m ^2^	YAM ≥ 70% and BMI ≥ 18.5 kg/m ^2^
n	107	157	18	65
Mean age (years)	85.5 ± 7.8	84.0 ± 7.6	79.5 ± 9.7	82.3 ± 7.5
Female sex: n (%)	83 (77.6)	121 (77.1)	11 (61.1)	43 (66.2)
Mean BMI (kg/m ^2^ )	16.66 ± 1.36	21.44 ± 2.91	16.37 ± 1.31	22.98 ± 2.49
Mean YAM (%)	55.0 ± 8.1	59.0 ± 7.6	73.8 ± 4.1	76.7 ± 8.0
Mean operative time (minutes)	52.6 ± 15.8	57.5 ± 19.4	54.4 ± 15.9	59.8 ± 20.8
Mean blood loss (mL)	111.8 ± 85.7	150.7 ± 122.3	120.2 ± 60.9	125.2 ± 75.8
Fracture: n (%)	1 (0.9)	4 (2.5)	0 (0.0)	3 (4.6)

Abbreviations: BMI, body mass index; YAM, young adult mean.

Patients were initially classified into 4 groups based on the combination of BMI (≥ 18.5 or < 18.5 kg/m ^2^ ) and YAM (≥ 70% or < 70%) ( Table 3 ). Intraoperative periprosthetic fractures occurred in 5.36% of patients with YAM ≥ 70% and BMI ≥ 18.5 kg/m ^2^ , 0.00% in those with YAM ≥ 70% and BMI < 18.5 kg/m ^2^ , 4.85% in patients with YAM < 70% and BMI ≥ 18.5 kg/m ^2^ , and 7.10% in those with YAM < 70% and BMI < 18.5 kg/m ^2^ .

**Table 3:** Intraoperative fracture rate by YAM and BMI

Group	N	Fractures (n)	Fracture rate (%)
YAM ≥ 70% and BMI ≥ 18.5 kg/m ^2^	112	6	5.36
YAM ≥ 70% and BMI < 18.5 kg/m ^2^	39	0	0.00
YAM < 70% and BMI ≥ 18.5 kg/m ^2^	268	13	4.85
YAM < 70% and BMI < 18.5 kg/m ^2^	169	12	7.10

Abbreviations: BMI, body mass index; YAM, young adult mean.

Stratified analysis according to BMI showed that in patients with BMI ≥ 18.5 kg/m ^2^ , no significant association was observed between YAM and intraoperative fracture (OR: 1.11, *p* = 0.80). In contrast, in patients with BMI < 18.5 kg/m ^2^ , no fractures were observed in those with YAM ≥ 70%, whereas fractures occurred exclusively in those with YAM < 70% (OR: 0.0, *p* = 0.128) ( Table 4 ).

**Table 4:** Stratified analysis according to the body Mass index (BMI)

BMI category	Odds ratio	*p* -value
≥ 18.5 kg/m ^2^	1.11	0.80
< 18.5 kg/m ^2^	0.0	0.128

Additionally, when patients were categorized into 4 groups based on YAM levels alone (≥ 70%, 60–69%, 50–59%, and < 50%), the fracture rates demonstrated a gradual increasing trend: 3.97%, 5.24%, 5.88%, and 6.58%, respectively. Although these differences did not reach statistical significance, a dose-dependent tendency was observed ( Table 5 ). Furthermore, among the 577 patients for whom operative time and blood loss data were available, patients with BMI ≥ 18.5 kg/m ^2^ demonstrated significantly longer operative time (60.5 ± 20.9 minutes versus 55.3 ± 16.8 minutes; *p* = 0.006) and greater blood loss (133.5 ± 100.4 mL versus 109.5 ± 76.4 mL; *p* = 0.004) compared with those with BMI < 18.5 kg/m ^2^ ( Table 6 ).

**Table 5:** Intraoperative fracture rate by detailed YAM categories

YAM group	N	Fractures (n)	Fracture rate (%)
≥ 70%	151	6	3.97
60–69%	191	10	5.24
50–59%	170	10	5.88
< 50%	76	5	6.58

Abbreviation: YAM, young adult mean.

**Table 6:** Comparison of operative time and blood loss according to the body Mass index (BMI)

BMI category	n (N = 577)	Mean operative (minutes)	Mean blood loss (mL)	*p* -value (operative time)	*p* -value (blood loss)
≥ 18.5 kg/m ^2^	372	60.5 ± 20.9	133.5 ± 100.4	0.006	0.004
< 18.5 kg/m ^2^	205	55.3 ± 16.8	109.5 ± 76.4		

In addition, multivariable logistic regression analysis adjusting for age, sex, operative time, and blood loss did not identify any independent predictors of intraoperative fracture; adjusted odds ratios for low BMI and low YAM were not statistically significant, likely due to the small number of events. Interaction testing (BMI < 18.5 kg/m ^2^ × YAM < 70%) also did not reach statistical significance. Post-hoc power analysis indicated that the observed difference (≈ 1% versus 0% fracture rate in the underweight group) provided only ∼ 12% power, whereas a clinically meaningful difference (6% versus 0%) would have yielded ∼ 49% power. Therefore, the lack of statistical significance likely reflects limited power rather than absence of association. Operative parameters differed significantly between BMI groups: patients with BMI ≥ 18.5 kg/m ^2^ had longer operative time (59.2 ± 19.3 versus 54.4 ± 15.5 minutes; *p* = 0.009) and greater blood loss (144.3 ± 116.2 vs 125.0 ± 93.1 mL; *p* = 0.024).

## Discussion

In contemporary management of proximal femoral fractures, early surgical intervention within 48 hours has consistently been associated with improved outcomes. Moja et al. 1 reported a pooled OR of 0.74 (95%CI: 0.67–0.81) for mortality when surgery was performed within 48 hours compared to delays, based on > 190 thousand patients. Similarly, another systematic review confirmed reduced 30-day and in-hospital mortality with early surgery. 2

Choice of implant also influences intraoperative fracture risk. Meta-analyses have found that cemented stems are associated with significantly fewer intra- and postoperative periprosthetic fractures compared to cementless options. 3 Registry data support a 59% relative risk reduction with cemented stems. 4

In our study, we observed a clear inverse trend between preoperative YAM and intraoperative fracture risk, especially in underweight patients (BMI < 18.5 kg/m ^2^ ), with a suggestive trend despite not reaching statistical significance. This indicates that YAM assessment may provide valuable risk stratification among low-BMI patients.

Body mass index is routinely available in clinical practice, and preoperative YAM evaluation in underweight patients could guide both surgical planning and prehabilitation, involving early osteoporosis treatment.

In contrast, among patients with BMI ≥ 18.5 kg/m ^2^ , fracture rates did not differ by YAM. Notably, our sub-analysis of 577 patients showed that patients with higher BMI had significantly longer operative times (60.5 ± 20.9 versus 55.3 ± 16.8 minutes; *p* = 0.006) and greater blood loss (133.5 ± 100.4 versus 109.5 ± 76.4 mL; *p* = 0.004) ( Table 5 ). This indicates procedural complexity, rather than bone quality, drives risk in this group. Conversely, streamlined exposure in lean patients may lead to quicker—but riskier—stem insertion in the context of low bone quality.

Given that plain radiographs and BMI are universally available, there is potential for artificial intelligence (AI)-driven prediction models to estimate YAM and forecast fracture risk, enhancing preoperative planning in frail patients.

Although the key comparison between patients with BMI < 18.5 kg/m ^2^ and YAM < 70% did not reach statistical significance ( *p* = 0.128), the divergent trend observed between low– and high–BMI groups in relation to bone mineral density is clinically meaningful. The lack of significance is most likely attributable to limited statistical power, particularly because the subgroup of patients with BMI < 18.5 kg/m ^2^ and YAM ≥ 70% was very small in our cohort. This highlights the need for larger multi–center studies or pooled analyses to confirm and strengthen these preliminary findings.

Moreover, a recent large cohort study demonstrated that the association of total hip BMD with incident hip fractures may be weaker among underweight individuals than among those with higher BMI, supporting our findings regarding increased fracture vulnerability in low–BMI, low–YAM patients. 5 A prospective analysis in diabetic patients similarly confirmed low BMI as an independent and powerful risk factor for fractures. 6 Systematic reviews have also challenged the protective notion of obesity in low trauma fractures, emphasizing the complexity of BMI–fracture relationships. 7 Finally, recent data suggest that suboptimal bone health—including low BMD and osteoporosis—is significantly associated with periprosthetic fractures and poor hip arthroplasty outcomes. 8 9

## Limitations

- This was a retrospective, single-center study conducted at our institution.
- Despite observing consistent trends, sample size limitations may have reduced statistical power. Larger, multicenter validation is necessary.
- Although our institution predominantly uses cementless hemiarthroplasty, a shift toward cemented stems is possible. Cementless fixation remains necessary in select patients, and external validation in varied cohorts is needed.

## Conclusion

In cementless hemiarthroplasty for femoral neck fractures among patients with BMI < 18.5 kg/m ^2^ , preoperative YAM evaluation can help reduce intraoperative periprosthetic fracture risk, highlighting the importance of bone quality assessment in surgical planning for underweight patients.
